# Community-oriented primary care footprinting: An undergraduate programme experience

**DOI:** 10.4102/safp.v66i1.5854

**Published:** 2024-04-18

**Authors:** Anastasia E. Ugwuanyi

**Affiliations:** 1 Department of Family Medicine and Primary Care, Faculty of Health Sciences, University of the Witwatersrand, Johannesburg, South Africa

**Keywords:** community-oriented primary care project, medical education, experiential learning, social accountability, Nelson Mandela Fidel Castro programme, University of the Witwatersrand

## Abstract

The Nelson Mandela Fidel Castro (NMFC) programme, a government initiative to address healthcare inequities in South Africa, focuses on the training of indigenous students to become competent healthcare practitioners. A collaboration combining training in a Cuban primary care, preventative system with integration in a South African institution within a quadruple disease burdened healthcare system. This article reflects on integration experience at the University of Witwatersrand, a programme pedagogically positioned within a workplace-based, situated learning framework. Since 2022, community-oriented primary care (COPC) projects became part of the integrated primary care and family medicine learning objectives. This article summarises the experience of the 2021–2022 cohort and calls for the strengthening of undergraduate medical education curricula with learning objectives reflective of social accountability.

**Contribution:**

This article spotlights work in the undergraduate space around teaching and experiential learning of community-oriented primary care in line with the journal’s scope.

## Introduction

The onset of the 21st century has heralded significant transformations in healthcare practice and education, reflecting global trends in health and disease. Notably, the Severe Acute Respiratory Syndrome coronavirus disease 2019 (COVID-19) pandemic’s public health response underscored the role of primary care and health promotion at both community and individual levels. This crisis accelerated the adoption of new learning pedagogies, aligning educational shifts with emerging paradigms in health professions education. The growing focus on integrating healthcare systems thinking into educational learning outcomes, underscores the importance of developing graduates whose competence align precisely with their training goals, encompassing knowledge, skills and professional competencies attuned to the dynamics affecting individual, family and community health.^1,2^

Many decades after Sidney and Emily Kark’s groundbreaking work at the Pholelea Health Centre, the concept of ‘community-oriented primary care’ (COPC) has emerged as a holistic approach to primary health care. Community-oriented primary care merges public health principles with primary care practices, addressing both individual and community health needs.^3^ This approach enriches health professions education by providing a contextually rich backdrop for training. The Nelson Mandela Fidel Castro programme, within the Department of Family Medicine and Primary Care at the University of the WItwatersrand, has pioneered integration of COPC projects within its undergraduate programme offerings within its integrated primary care (IPC) curriculum. This initiative provides students with vital experiential learning opportunities, crucial in the contemporary healthcare environment. These experiences are grounded within the communities and families they serve, embodying Lave and Wenger’s situated learning theory.^1^

At the core of community-oriented primary care, is a comprehensive approach to patient care, which includes community involvement in both treatment and disease prevention. As healthcare needs evolve, particularly in underserved areas, COPC’s role in facilitating equitable and effective care becomes increasingly important.^3^ Its introduction at the undergraduate level is essential, ensuring that future healthcare providers understand and value its significance in context. The COPC effectively bridges the gap between traditional facility-based services and the real contexts of family and community life.^4,5^

**FIGURE 1:**
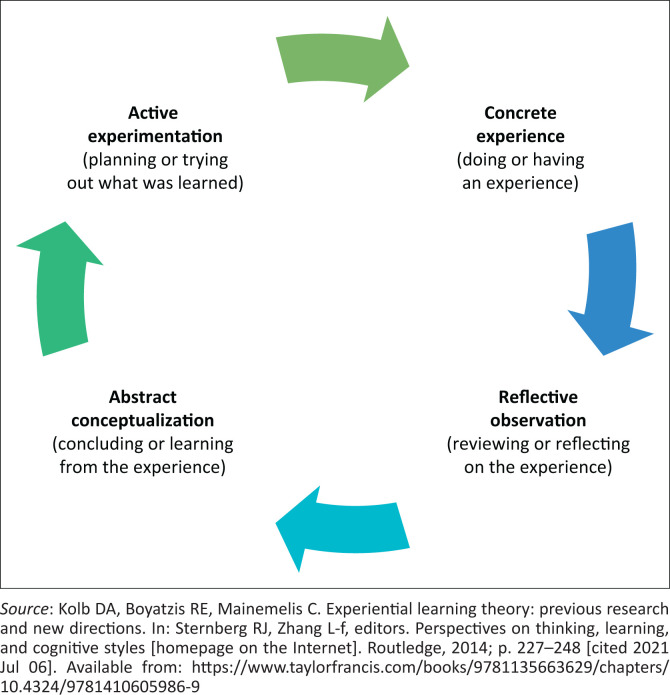
Kolb’s experiential learning cycle.

Modern medical education aims to equip graduates with diverse skills, from clinical acumen to effective communication and critical thinking. The COPC enhances these capabilities by offering a practical application of theoretical knowledge, thus sharpening the practical skills of medical students.^3,6^ Following Kolb’s experiential learning theory (Figure 1)^7^, COPC fosters a cycle of learning: engagement with community health workers and patients in the community (experience), reflective analysis of these interactions (reflection), and application of this learning in practical settings (application). Such a cycle is fundamental to contemporary medical training.^7,8^

## The Nelson Mandela Fidel Castro Programme Exemplar

In 2022, the WITS-NMFC (The University of the Witwatersrand-Nelson Mandela Fidel Castro) collaboration embarked on an ambitious project to integrate COPC into its medical education curriculum. This initiative involved the fourth cohort of 150 students, distributed across five academic health complexes (AHCs) in Gauteng and the North West. The aim was to provide students with a deeply immersive learning experience, facilitated by family physician preceptors and clinical educators across these AHCs. The programme sought to not only impart knowledge but also instil a profound understanding of medical practice in diverse socioeconomic environments through a community-oriented approach.

Reflective feedback from the 2021–2022 cohort highlighted the transformative impact of engaging in community-oriented primary care. Students reported that interactions with individuals and families within their geosocial contexts profoundly influenced their perception of medical practice. This exposure provided them with a genuine ‘social perspective of learning’, aligning with the transformative learning goals of the programme and reshaping their understanding of the social determinants of health.

## The Nelson Mandela Fidel Castro-WITS community-oriented primary care pedagogical background

The programme’s approach is grounded in a longitudinal clerkship model within IPC and family medicine. This model promotes situated learning, where students actively engage in primary health care settings under the supervision of family physicians and working alongside ward-based outreach teams. The curriculum involves comprehensive COPC activities, including the knowledge and application of formulating community diagnoses, and critical analysis of health determinants based on societal, geographical and economic factors. Students are trained to develop and implement health promotion initiatives aimed at improving health equity indicators across various communities.^1,9^

Integrating theories of workplace-based and situated learning, the programme embeds the community-oriented primary care project into a year-long curriculum focused on primary care. This includes a broad spectrum of health topics such as women’s health, encompassing reproductive health, antenatal care, and primary obstetric and gynaecological consultations. These are particularly relevant given South Africa’s human immunodeficiency virus (HIV) and tuberculosis (TB) burden, the prevalence of teenage pregnancies and contraception uptake. Mental health, gender-based violence and intimate partner violence are also addressed in the primary care context. The programme further incorporates children’s health, emphasising the integrated management of childhood illness (IMCI) approach, which links primary care to tertiary healthcare systems. It covers the management of acute and chronic conditions in primary care settings, introduces interprofessional practice, and examines health system dynamics and their impact on individual and community health indices.

## Analysing the learning experience within an experiential learning theories framework

The DFMPC at the University of the Witwatersrand demonstrates a profound commitment to experiential learning in its University of the Witwatersrand model of incorporating community level engagement by the students. Within a framework of communicated objectives, the process commenced with students (150 in 25 small groups) assimilated into 5 AHCs, interacting with communities, families, of 25 key patients across a network of community health centres (CHCs) and primary health care (PHC). These experiences provided a robust foundation for interdisciplinary engagements and collaborations, immersing students in the practical aspects of healthcare.

In demonstration of the reflective observation stage of Kolb’s experiential learning cycle, students participated in group work as they mapped their chosen communities, identified, and interviewed relevant stakeholders as well as interviewed families of the key patient. These engagements were guided by the objectives under the supervision of clinical educators and family physicians. Their collaboration with ward-based outreach teams (WBOTs) and engagement with interprofessional networks allowed for a tangible connection with the community. This reflective phase was critical as students contextualised their on-the-ground experiences with their academic knowledge, pondering the complex socioeconomic determinants of health they witnessed.

The abstract conceptualisation phase was marked by the assessment of group assignments by a team led by family physicians and community practitioners. These assessments, which contributed to the IPC year marks, provided an opportunity for students to transform their practical experiences into academic learnings and conceptual understanding.

The cycle culminated in active experimentation, with students applying their insights into practical settings, testing theories and intervention strategies within the community. This final transformative stage, where learning was realised through direct application, was an essential part of preparing the students for their eventual roles as medical practitioners. This comprehensive process was in alignment with Kolb’s experiential learning theory.^8^

## Nelson Mandela Fidel Castro-WITS community-oriented primary care assignment objectives

Developed on the ‘Plan-Evaluate-Implement’ cyclical iteration of COPC as a health systems intervention,^8^ the programme’s communicated and assessed objectives are summarised in Table 1. As an undergraduate learning activity, 2023 will be the second year of engaging the students in a community-oriented primary care project.

**TABLE 1:** Summary table showing alignment of outcomes with community-oriented primary care objectives.

COPC programme objectives	Students’ demonstration of active learning outcomes
To profile a chosen community and develop a provisional community diagnosis with the data and experience of community health workers and other allied healthcare professionals deployed in the community.	This was successfully performed by each group at varying levels of depth and subject matter understanding. Groups from the Sedibeng AHC received in-depth tutorials from a COPC-experienced family physician and as such were able to present community diagnosis that detailed every possible environmental determinant affecting health.
To explore the challenges of intersectoral collaboration and community participation of identified healthcare and non-healthcare stakeholders and the effect it has on the community health profile.	Intersectoral engagements with clinical managers, WBOT teams and family physicians differed across the various AHCs expectedly and this depended on the organisational structure and burden of work, appropriate oversight and competence of the community health workers, similar to the experience described by a family physician in reviewing NHI readiness of a comprehensive healthcare practice and following the format of an abbreviated Local Institutional Support Assessment (LISA).^8,9^
To describe a patient and link their biopsychosocial assessment to problems identified in the community diagnosis, and their management using family medicine management frameworks.	Student groups were expected to reflectively use family medicine tools such as an Ecomap and a Genogram in describing the family and ecological environments defining the health or ill-health of their profiled patients. Variations in understanding of the appropriate use of such tools also showed up in the presentations, demonstrating the COPC project as an authentic learning and assessment methodology of undergraduate family medicine education.
To reflect on health promotion and communication in the context of the index patient’s problems and related problems in the family and the community.	This objective was interrogated as students demonstrated varying levels of understanding of health promotion as a concept and the contextualisation within their chosen COPC project, reflexively reported in their presentations and written portfolio.
To reflect as a group on the impact that the COPC project had on future practice and current learning.	It was very gratifying to see and hear how student groups who embraced the experience of the COPC project reflected on their projections of future practice from a community-oriented perspective during their panel presentations.

AHC, academic health complexes; COPC, community-oriented primary care; WBOT, ward-based outreach teams; NHI, National Health Insurance.

**FIGURE 2:**
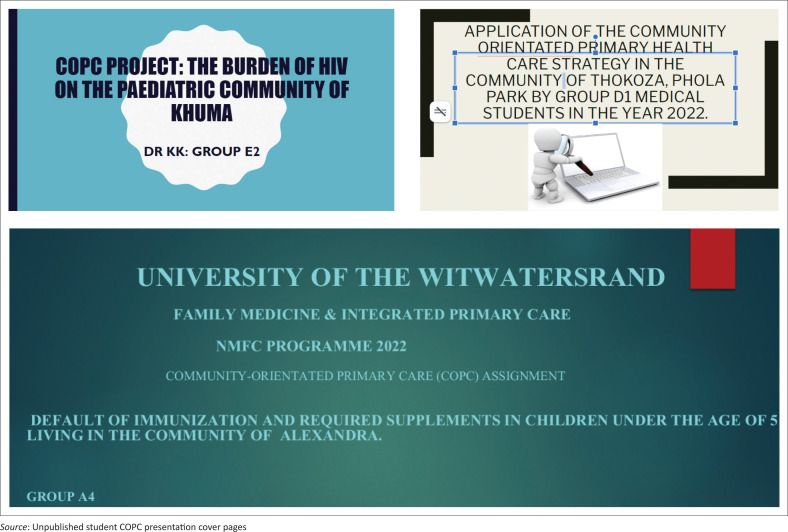
Some examples of the community-oriented primary care student group project presentations.

**FIGURE 3:**
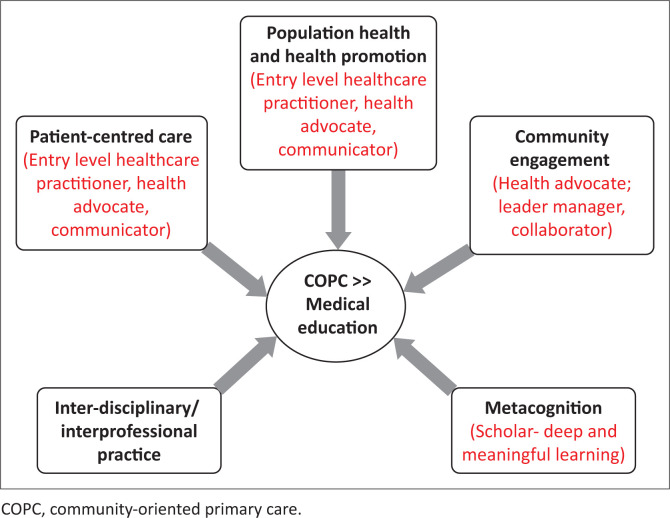
Encouraging graduate attributes through the implementation of community-oriented primary care undergraduate medical education activities. The WITS-Nelson Mandela Fidel Castro overarching objectives.

Figure 2 shows some of the project presentation front pages confirming the reality of the student’s experiences within actual communities in the different AHCs. The COPC project’s capacity to engender different family physician ‘graduate attributes’ is captured in Figure 3. The COPC experiential learning pedagogy employed within the WITS-NMFC programme showcases an innovative approach to medical education. Scaffolded on the framework of the experiential learning cycle by Kolb, the project iteration begins with concrete experience: 150 students are integrated into 5 AHCs, engaging with over 25 communities, 500 > families, and more than 25 key patients across various CHC and PHC facilities. This rich environment facilitates multiple interdisciplinary engagements and collaborations, allowing students to immerse themselves in the realities of healthcare provision and maintenance on the coal face of families in community.

Reflective observation is encouraged through the summative group work projects, guided by clinical educators and family physicians. Students work closely with WBOT and benefit from interprofessional linkages that offer genuine community level interface. This stage of reflective observation is crucial as students process their experiences, compare them to theoretical knowledge, and reflect on the socioeconomic determinants of health they encounter within the provided project time frame. This is then reported as a submission for assessment at the end of the year and presented to a panel for evaluation.

Abstract conceptualisation is facilitated during written and verbal presentation of group assignments at the end of the year, to a team comprised of family physicians and community practitioners. This assessment, which contributes to the students’ IPC year marks, allows students to project their practical experiences into viable and feasible interventions thereby aligning COPC practice with theory.

Finally, active experimentation is undertaken as students apply their newfound insights to real-world scenarios. With the support of the WBOT teams and family physicians, different groups applied low-scale strategies within the community settings. This transformative process, where learning is actualised through practice, is integral to preparing students for their future roles as medical practitioners, aligning with Kolb’s experiential learning theory.^7^

## The bigger picture

Department of Family Medicine and Primary Care, in the School of Clinical Medicine at the University of the Witwatersrand has successfully entrenched COPC learning objectives into all its undergraduate programmes. This integration reflects the department’s commitment to inculcating deep, meaningful learning objectives and realising the significant social impact of medical education. Each division within the DFMPC employs its unique approach to teaching, learning and assessing COPC, yet there is a shared understanding of the importance of embedding social justice principles in learners. This unity amidst diverse pedagogical methods underscores the essential role of COPC in shaping future medical professionals.

One emerging area of research is the evaluation of the transformative effects of these COPC projects on the communities where students engage in situated learning. In the long term, research will focus on assessing how these experiences influence the professional development of students as they transition into practitioners. Policy-level goals include aligning COPC’s educational objectives with broader public health initiatives, such as the National Health Insurance scheme, addressing issues like migration, global health dynamics and the use of Artificial Intelligence (AI) in public health to enhance community healthcare indices.^10^

The DFMPC’s initiative of incorporating COPC projects into its curriculum is a pioneering step towards fostering professional capacity for integrated care, beginning at the undergraduate level. Feedback from the 2021–2022 cohort highlights the profound impact of this approach. Exposure to COPC provides undergraduates with a genuine and invaluable learning experience, equipping them to address the real healthcare challenges faced by communities in South Africa. These experiences are not just academic exercises; they are essential preparations for dealing with the complexities of real-world healthcare scenarios.

## References

[1] O’Brien BC, Battista A. Situated learning theory in health professions education research: A scoping review. Adv Health Sci Educ. 2020;25(2):483–509. 10.1007/s10459-019-09900-w 31230163

[2] Faculty of Medicine and Health Sciences, Stellenbosch University. Graduate attributes [homepage on the Internet]. Faculty of Medicine andHealth Sciences; 2013 [cited 2023 Jul 28]. Available from: https://www.sun.ac.za/english/faculty/healthsciences/Documents/Graduate%20attributes%20FMHS%20-%20ENGLISH%20-%201%20July%202013.pdf

[3] Tollman SM. The Pholela Health Centre – The origins of community-oriented primary health care (COPC): An appreciation of the work of Sidney and Emily Kark. SAMJ. 1994;84(10):653–658. 7839251

[4] Frenk J, Chen L, Bhutta ZA, et al. Health professionals for a new century: transforming education to strengthen health systems in an interdependent world. Lancet. 2010;376(9756):1923–1958. 10.1016/S0140-6736(10)61854-5 21112623

[5] Institute of Medicine (US) Division of Health Care Services, Connor E, Mullan F. Community oriented primary care: Meaning and scope. In: Community oriented primary care: New directions for health services delivery [homepage on the Internet]. National Academies Press (US); 1983 [cited 2023 Aug 07]. Available from: https://www.ncbi.nlm.nih.gov/books/NBK234632/25121320

[6] Community Oriented Primary Care (COPC). University of Pretoria. [cited 2023 Jul 25]. Available from: https://www.up.ac.za/family-medicine/article/2080699/implementing-copc-

[7] Kolb DA, Boyatzis RE, Mainemelis C. Experiential learning theory: previous research and new directions. In: Sternberg RJ, Zhang L-f, editors. Perspectives on thinking, learning, and cognitive styles [homepage on the Internet]. Routledge, 2014; p. 227–248 [cited 2021 Jul 06]. Available from: https://www.taylorfrancis.com/books/9781135663629/chapters/10.4324/9781410605986-9

[8] Moosa S. Community-oriented primary care for National Health Insurance in South Africa. Afr J Prim Health Care Fam Med. 14(1):e1–e4. 10.4102/phcfm.v14i1.3243 PMC8905368 35261262

[9] Marcus T, Hugo J. Community-oriented primary care: Where there is a doctor. In: Mash B, editor. Handbook of family medicine. 4th ed. OUP Southern Africa: Oxford University Press, 2017; p. 390–416.

[10] Matmi MM, Alnonazi AE, Sulaimani AM, et al. Application of artificial intelligence in community-based primary health care: Systematic review. J Namib Stud Hist Polit Cult. 2023;35:1269–1292.

